# Associations Between Sickle Cell Disease, Pica, and Enuresis in Pediatric Neurodevelopmental Disorders

**DOI:** 10.3390/medsci14020186

**Published:** 2026-04-07

**Authors:** Kit Neikirk, Aliyah Allick, Christopher J. Gamper, Alicia D. Cannon, Wilfreda Lindsey, Bridget G. Gibbons, Eboni I. Lance

**Affiliations:** 1 Department of Neurodevelopmental Medicine, Kennedy Krieger Institute, Baltimore, MD 21205, USA; 2 Department of Pediatrics, The Johns Hopkins University School of Medicine, Baltimore, MD 21205, USA; christopher.gamper@regeneron.com; 3 Department of Neuropsychology, Kennedy Krieger Institute, Baltimore, MD 21205, USA; cannona@kennedykrieger.org; 4 Department of Neurology, The Johns Hopkins University School of Medicine, Baltimore, MD 21205, USA; 5 Department of Behavioral Psychology, Kennedy Krieger Institute, Baltimore, MD 21205, USA

**Keywords:** pica, enuresis, pediatrics, sickle cell disease, health disparities

## Abstract

**Background**: Sickle cell disease (SCD) is a hereditary disorder affecting red blood cells’ shape and functional capacity. Individuals with SCD report relatively high co-occurrence of neurodevelopmental disorders (NDDs). In addition, these children also have higher rates of enuresis (incontinence) and pica, disorders prevalent in children with developmental delays. Both enuresis and pica can have negative effects on mental health, but their pathophysiology, especially in SCD, remains unclear. **Objectives**: The objective of this study was to determine the rates of pica and enuresis in a pediatric SCD clinic to compare the co-occurrence of NDDs and enuresis/pica. **Methods**: To do so, we performed a cross-sectional explanatory retrospective chart review of 275 pediatric SCD patients. **Results**: Our SCD cohort had a 27% prevalence of enuresis, 9% prevalence of pica, and 24% prevalence of one or more NDDs. We noted significant inter-group overlap between pica/enuresis and other risk SCD severity factors. NDDs were approximately twice as frequent in SCD patients with pica or enuresis compared to those without. While pica was associated with HbS*β*^+^, it was not linked to disease severity indicators. Enuresis was associated with hydroxyurea usage (66.7% vs. 42.6%, *p* = 0.001) and reticulocyte counts, indicative of higher disease severity. **Conclusions**: Clinically, these results are the first to show co-occurrence between pica, enuresis, and NDDs in SCD. We suggest that the occurrence of pica or enuresis may serve as an indicator for previously unknown NDD risk. Together, these results underscore the need for targeted screenings of pica and enuresis in SCD populations.

## 1. Introduction

Sickle cell disease (SCD) is a hereditary disorder that affects the shape and functional capacity of red blood cells, leading to vaso-occlusive events resulting in complications including chronic pain, anemia, and increased risk of infections [1,2,3]. In the United States, per the Centers for Disease Control, approximately 100,000 people are affected by SCD, and it is particularly prevalent in Black and African American individuals [4]. The term SCD encompasses at least 13 genetically distinct hemoglobinopathies and heterozygous variants, including homozygous HbSS and HbS/β-thalassemia, each producing differing degrees of hemolysis, vaso-occlusion, organ injury, and clinical severity [5]. In all subtypes, significant complications include cerebrovascular disease and cognitive impairment [2], which can lead to central nervous system involvement.

Individuals with SCD have higher rates of neurodevelopmental disorders (NDDs), including attention deficit hyperactivity disorder (ADHD), intellectual disabilities (ID), and specific learning disabilities [3,6]. As previously reviewed by Schatz & McClellan [7], while the exact effects of SCD on the central nervous system remain unclear, epidemiologically, approximately between a quarter and a third of children with SCD experience concomitant neurodevelopmental complications [8]. Although NDDs are associated with both mental health concerns and SCD-related complications, knowledge about associated risk factors for NDDs in SCD remains limited. The objective of this study was to determine the impact of SCD on two conditions known to be associated with NDDs: enuresis and pica.

Enuresis, or incontinence, is a prevalent issue that the International Children’s Continence Society defines as occurring until a cutoff age of 5 years old [9]. Enuresis diagnoses may further be separated into primary enuresis, defined as children who have never been continent for over 6 months, and secondary enuresis, defined as children with relapse after over 6 months of continence [10]. Cumulatively, these diagnoses affect up to 5–7 million children (equivalent to approximately 5–10% of the general population) in the United States [11]. Nocturnal enuresis, defined as nighttime incontinence at least two times weekly, is associated with reduced self-esteem and social isolation [12]. Nocturnal enuresis more commonly occurs among people with SCDs [12]. Male sex is a major risk factor for enuresis in individuals of all ages with and without SCD, with reported rates as high as 50% of boys with homozygous SCD [13]. Additional risk factors for enuresis in SCD include younger age, thalassemia major, sleep-disordered breathing, and NDDs [12,14]. Regardless of frequency and etiology, enuresis significantly impacts quality of life. In children with SCD, increased nocturnal enuresis is associated with higher reported psychosocial problems, as determined by the Pediatric Symptom Checklist [15]. Additionally, a recent study found that poorer health-related quality of life, disrupted sleep, increased self-reported fatigue, and higher disease severity markers, such as elevated lactate dehydrogenase, are all risk factors for enuresis in SCD [16]. Contrastingly, enuresis has also been correlated with higher fetal hemoglobin, which often signifies improved clinical outcomes in SCD, yet this association may be mediated by increased hydroxyurea in both cases, despite hydroxyurea’s urine-concentrating effect [16,17]. Although the 2019 American Society of Hematology guidelines recommend that the enuresis status be discussed as a screening tool for polysomnography [18], enuresis screenings in the context of SCD lack standardization. Standardization is hampered in part due to the unclear etiology and disease risk factors for enuresis, highlighting the need to clarify the prevalence and implications of enuresis in SCD.

Another significant issue observed in both NDDs and SCD is pica [19,20]. Pica is broadly defined as the compulsive ingestion of non-digestible or non-nutritive substances—such as dirt, wood, or soap—over a period of at least one month [19,20]. Pica is commonly reported in individuals with NDDs, such as ASD and ID [21], as well as patients with SCD [20]. While pica’s pathophysiology remains unclear, especially in the context of SCD, prior research has shown a high prevalence, ranging from 5% to 30% of children, with a higher prevalence in SCD patients than in the general population [22,23]. Among SCD patients, younger individuals and those with homozygous SCD have higher rates of pica compared to older individuals or those with heterozygous SCD [22]. Another study of a majority-white population with SCD in Belgium found similar results; researchers observed no differences dependent on sex but noted that individuals reporting pica were significantly younger and had lower median hemoglobin levels [24]. Despite the relatively high prevalence of pica, like enuresis, many screening techniques are limited. A past chart review of pica indicated that although 21% to 34% of patients displayed behaviors emblematic of pica, screening occurred less than 35% of the time [25]. Like enuresis, pica can have neurodevelopmental, medical, and psychosocial implications, making it important to discuss in standard visits [26]. In the general population, pica can cause substantial burdens on the family, including embarrassment and significant health risks, such as bowel obstruction and damage to teeth [27]. The unclear etiological mechanisms of pica highlight the importance of exploring the interdependence of this condition with NDDs and SCD severity.

It is well-established that NDDs, including ASD and ID, are associated with enuresis [28,29] and pica [21], but associations between these disorders and SCD have not yet been considered. Furthermore, it remains unclear if in SCD patients with NDDs, pica and enuresis more commonly occur. Thus, the objective of this study was to determine the rates of pica and enuresis in a local pediatric SCD clinic. Additionally, we explored whether these conditions have different disease markers or associations in SCD compared to the general population. To do so, grounded in substantial literature [30,31,32], we performed a retrospective chart review. Given that NDDs frequently occur concomitantly with SCD [33], it was hypothesized that enuresis and pica would display a relatively high prevalence in this pediatric population, accompanying NDD diagnoses.

## 2. Methods

### 2.1. Cohort

Inclusion criteria for this study encompassed patients under 18 years old at the time of initial database creation, with hematology/laboratory-confirmed diagnoses of SCD. Exclusion criteria included incomplete charts and patients under 5 years old (for purposes of enuresis diagnosis). We obtained patient information from the clinic rosters of two local sickle cell disease and neurodevelopmental clinics in a Northeastern United States metropolitan area. We then accessed records through the Epic Hyperspace system [34]. Both institutions are urban pediatric hospitals and ambulatory outpatient care centers, sharing a single charting system, which was accessed.

### 2.2. Chart Review

**Figure 1:**
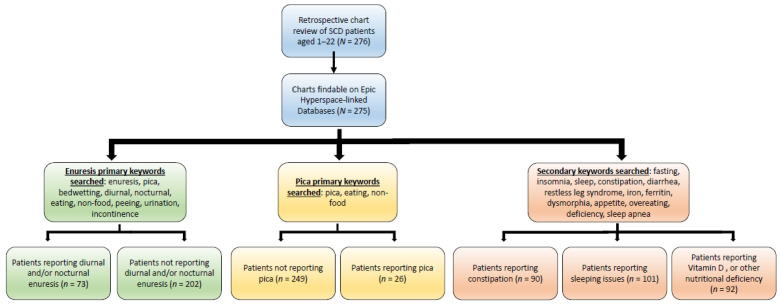
Flow chart of retrospective chart review performed. Abbreviations: SCD, sickle cell disease.

We utilized a chart review from a previously collected pediatric medical record that contains extensive information about SCD patients, many of whom have concomitant NDDs [33]. Charts of 340 patients were reviewed for the above inclusion criteria, with 275 charts qualifying for additional review/data extraction (Figure 1). The chart years contained within the database differed on a per-patient basis. All searching and accessing of records was performed in 2023 across two months in a cross-sectional manner. The database included demographic (e.g., age and sex), medical, and educational history, as well as disease information such as SCD genotype, SCD treatment history (e.g., hydroxyurea, chronic transfusions), and history of disease complications/comorbidities (e.g., stroke, acute chest syndrome, pain crisis, splenic sequestration, infection, and dactylitis). Additionally, this database included information pertinent to NDDs, including neurodevelopmental diagnoses (e.g., ADHD, attention issues, developmental delay, specific learning disabilities in math and reading comprehension, language disorders, and anxiety), psychiatric diagnoses associated with neurodevelopmental disorders (e.g., anxiety, depression, etc.), and school services [e.g., Individualized Educational Plan (IEP), 504 Rehabilitation Plan (504 Plan)].

Charts were searched for keywords related to pica (associated terms queried: pica, eating, non-food, malnutrition, and eating disorder) and enuresis (associated terms queried: enuresis, enuretic, nocturnal, diurnal, peeing, urination, and incontinence). Criteria for pica diagnosis required the physician to note in the charts the eating of non-food items or pica. Criteria for enuresis diagnosis required a physician’s notes in the charts mentioning bedwetting or enuresis. In both cases, therefore, diagnosis was dependent on a physician’s reporting of pica or enuresis. We also expanded the database to explore the paradigms of sleeping disorders (i.e., reported insomnia or sleep apnea), constipation, or nutritional deficiencies (associated terms queried: fasting, insomnia, sleep, constipation, diarrhea, restless leg syndrome, iron, ferritin, dysmorphia, appetite, overeating, deficiency, and sleep apnea). Finally, recent patient lab values (i.e., hemoglobin, fetal hemoglobin, mean reticulocyte count, mean white-cell count, mean platelets, mean ferritin serum, mean transferrin, mean iron serum, mean TIBC, and mean iron saturation) were accessed, if available. For lab values, the most recent three lab values were averaged for each patient. If fewer than three lab values were available, lab values were not included.

### 2.3. Statistical Analyses

Data were collected using Microsoft Excel 2023 and exported into Stata IC-13 (StataCorp, College Station, TX, USA) for analyses. Logistic regression analyses were used with specific neurodevelopmental diagnoses as the outcome variables, as indicated in the Figure Legends. Chi-squared test, with additional post hoc test, was performed to calculate residuals for nominal variables. The Wilcoxon rank-sum test was used as the non-parametric equivalent to a *t*-test for all comparisons of interval variables. A *p*-value of ≤0.05 was accepted for significance.

### 2.4. Ethics Approval

All study procedures were reviewed and approved by the Institutional Review Board (Parent ID IRB00087766; Protocol CR00053361) under an expedited review pathway.

## 3. Results

### 3.1. Co-Occurrence of Pica and Enuresis with NDDs

**Figure 2:**
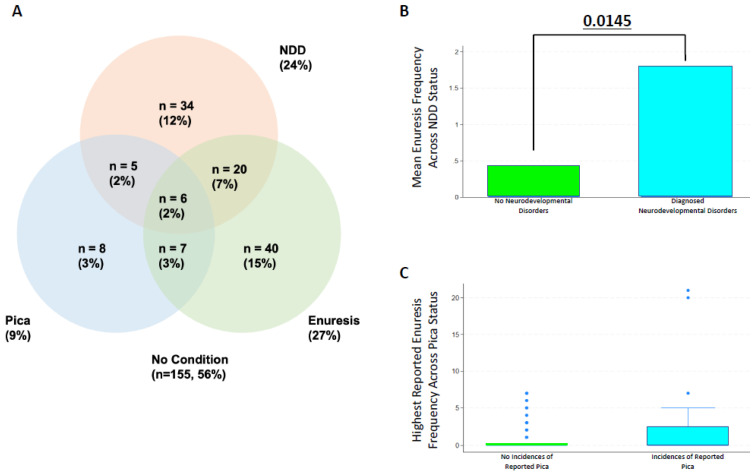
(**A**) Venn diagram showing the co-occurrence of NDDs, enuresis, and pica in SCD. (**B**) The mean enuresis frequency reported among those with and without NDDs. (**C**) The highest reported enuresis frequency, if available, reported among those with and without pica. *p*-value is presented above with statistical analysis performed via the Wilcoxon rank-sum test. Abbreviations: NDD, neurodevelopmental disorder.

To begin, we examined the co-occurrence of NDDs with pica and enuresis. Consistent with past results [33], 24% of patients (*n* = 65) had an NDD diagnosis. Nine percent of patients (*n* = 26) had pica, and 27% (*n* = 73) had enuresis. Most patients without pica and with enuresis reported nocturnal enuresis only. Enuresis and pica each showed significant associations with NDD occurrence (*p* = 0.005 and *p* = 0.019, respectively). Fourteen percent of the study cohort also had two or more of the conditions: pica, enuresis, or NDDs (Figure 2A). Specific NDDs, including global development delays (11.0% vs. 2.5%, *p* = 0.003) and specific learning disabilities (SLD), in math (6.9% vs. 2%, *p* = 0.045) were significantly more likely to be diagnosed in individuals with enuresis than those without. However, these differences in developmental delays did not confer a significant difference (*p* = 0.108) in rates of school support services (e.g., IEP or 504 plan). Among individuals with pica, language disabilities occurred at a significantly higher rate (19.2% vs. 7.6%, *p* = 0.046). Yet, school support services were not significantly associated with pica status (*p* = 0.514). Together, 44% of patients had either an NDD, pica, or enuresis, with these statuses frequently occurring alongside one another.

From there, we sought to understand if the co-occurrence of enuresis, pica, and/or NDDs increased the relative severity of these conditions. When we stratified enuresis frequency (as reported in incidents per week by physicians, if available within the charts) by NDD status, individuals with NDDs had significantly higher frequencies of reported enuresis (Figure 2B). Additionally, patients with both enuresis and pica had higher enuresis incident frequency than patients without pica (Figure 2C), but results were not statistically significant, likely due to the small sample size of pica. Together, this shows an interdependence between pica, enuresis, and NDDs, with pica and/or NDDs increasing the occurrence of enuresis. From there, we sought to define the characteristics of individuals with enuresis and pica.

### 3.2. Sickle Cell Disease Enuresis Characteristics Analyses

In our study, 27% (*n* = 73) of patients’ charts yielded either current or past reports of enuresis (Figure 2A), mostly nocturnal only (*n* = 58, 21%) or both nocturnal and diurnal (*n* = 7, 2.5%), with a small proportion either being unspecified (*n* = 7, 2.5%) or diurnal only (*n* = 1, 0.3%). Beyond associations with NDD, enuresis was not significantly associated with age (9.12 vs. 8.57 years among those with and without enuresis, respectively; *p* = 0.365). While males had a slightly higher prevalence of enuresis compared to females, this was not statistically significant [30.4% among males versus 22.6% among females, *p* = 0.143]. SCD genotype was also associated with enuresis, with homozygous-SS type SCD more common in patients with enuresis [76.7% rate among enuretic individuals vs. 56.9% rate among non-enuretic individuals; *p* = 0.003]. Interestingly, enuresis occurred less in patients with SC-type SCD compared with the non-enuretic population (9.6% vs. 31.7%, *p* = 0.0002). Patients who reported enuresis, as compared to those without enuresis, had a higher likelihood of stroke history (17.8% vs. 9.9%, *p* = 0.046), specifically silent cerebral infarct (13.7% vs. 4%, *p* = 0.004), as well as headaches (65.8% vs. 49%, *p* = 0.014). Enuresis was not statistically linked to constipation, diarrhea, or other sickle cell complications.

**Figure 3:**
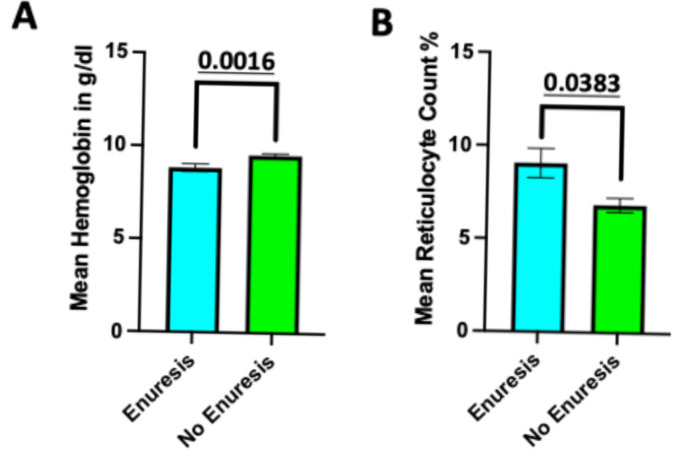
(**A**) Lab values of hemoglobin and (**B**) mean reticulocyte count (%) in SCD patients with and without enuresis. Median ± SD lab values represent the last 3 available labs for the patient (*n* = 3). *p*-value is presented above with statistical analysis performed via the Wilcoxon rank-sum test.

We also found associations between enuresis and SCD treatments, sleeping issues, and lab-measured hemoglobin levels and reticulocyte counts (Table 1; Figure 3A,B). Specifically, individuals with enuresis had statistically significantly lower median hemoglobin and higher mean reticulocyte counts, indicating higher disease severity (Figure 3A,B). We further found that patients with enuresis were significantly more likely to have previously been on disease-modifying therapies, such as hydroxyurea, than non-enuretic individuals (66.7% vs. 42.6%, *p* = 0.001). Given concerns that this finding may be due to the higher rates of enuresis in individuals with SS-type SCD, who are also more likely to be offered/on disease-modifying therapies, we repeated analyses including only SS-type SCD patients, with results continuing to be significant (81.8% vs. 66.1%, *p* = 0.034). This shows that in our population, enuresis was independently associated with disease severity.

### 3.3. Sickle Cell Disease Pica Characteristics Analyses

We found no significant demographic differences (e.g., sex and age), SCD-related complications, and lab values between patients with SCD with and without pica (Table 2). While patients reporting pica were slightly younger (7.54 years versus 8.84 years) and had a lower BMI (16.6 kg/m^2^ versus 17.8 kg/m^2^), neither of these differences was statistically significant. Additionally, within our database of other medical diagnoses (e.g., psychiatric, as described in Section 2), no other statistically significant correlates were present with pica. Notably, we found associations with S-β^+^ thalassemia type SCD [*n* = 6 (23.1%) with pica versus 21 (8.4%) without pica; *p* = 0.017)], but the overall distribution of genotypes among patients with and without pica was not significantly different (*p* = 0.051). Additionally, no SCD complications, including strokes (*p* = 0.477), pain crises (*p* = 0.855), and headaches (*p* = 0.649), vitamin deficiencies (*p* = 0.126), SCD treatments (*p* = 0.452), or lab values of blood and iron metrics were associated with pica status. This underscores that pica is relatively independent of other SCD and NDD risk factors within our patient population, outside of SCD subtype.

## 4. Discussion

### 4.1. NDDs in Sickle Cell Disease

Our retrospective chart review suggested a co-occurrence of pica and enuresis in SCD, potentially arising due to the association of both conditions with NDDs. This co-occurrence underscores the need for increased targeted screening and intervention strategies for NDDs.

### 4.2. Enuresis

Compared with other studies in SCD populations, our chart review found a lower prevalence of enuresis. Past studies have found rates of enuresis ranging from a common range of 38–52% in homozygous SCD [35]. In general, the prevalence of enuresis, even in SCD, decreases with age [36]. However, we found the average age of individuals with enuresis was slightly higher than that of their counterparts without enuresis. This may be due to having a study cohort composed of relatively older patients (mean age = 8.7 years) or because younger individuals were automatically disqualified from enuresis diagnosis in our study per the International Children’s Continence Society cutoff age of 5 years old [9]. As many providers did not report them separately, our study could not discriminate between primary and secondary enuresis. Relative rates of primary and secondary enuresis may have similar etiology and should be investigated in the future [37].

Our data was consistent with prior studies demonstrating that enuresis is associated with hydroxyurea usage [16]. Patients on disease-modifying therapies, like hydroxyurea, were more likely to have enuresis in our study, findings that persisted even when only SS-type SCD patients were included. Of note, enuresis is not a reported side effect of hydroxyurea, and hydroxyurea is associated with improved sleep in SCD [17]. This finding may be related to associations between hydroxyurea and worsening disease severity.

In line with previous findings [13], we found that homozygous SCD was more likely to be associated with enuresis (32.7% within-group prevalence of enuresis) than SC-type SCD (9.8% within-group prevalence of enuresis). Additionally, our findings of higher mean reticulocyte and lower mean hemoglobin count in individuals with enuresis suggest increased SCD severity, which may be independent of other SCD-associated complications (e.g., seizures and pain crises) [38]. They also displayed high rates of Global Developmental Delays and SLD for math, yet the relatively small subgroup sizes make it difficult to determine if these findings are simply secondary to the occurrence of NDDs in enuresis.

While there are sex differences noted in the general population with enuresis, sex-dependent differences were not found in our SCD sample. A previous study in Philadelphia showed increased rates of nocturnal enuresis in male adolescents with SCD, as compared with their female counterparts [39]. Another study suggested that sex-dependent differences arise in part due to blood composition; enuresis more commonly occurs in males with low concentrations of fetal hemoglobin and females with high mean corpuscular hemoglobin [13]. Our study did not find any significant differences in fetal hemoglobin between children with and without enuresis.

### 4.3. Potential Etiology of Enuresis in SCD

Sleep issues were associated with enuresis in our sample cohort. Nearly half of all individuals with sleep apnea also reported enuresis, aligning with previous findings that enuresis severity is associated with obstructive sleep apnea [40]. Prior findings also show that hyposthenuria, sleep-disordered breathing, and neurological dysfunction are each associated with and potentially causative of enuresis [12], and that enuresis in patients with SCD is more severe and long-lasting than in patients without SCD [41]. Past results indicate that blood–brain barrier (BBB) disruption can occur with a high severity of SCDs [42]. Increased BBB permeability results in the infiltration of innate immune cells and neuroinflammation [43]. The entry of these inflammatory mediators into the central nervous system may affect brain regions involved in autonomic control, thereby dysregulating bladder function [43]. Additionally, sleep dysfunction is associated with autonomic dysfunction [44]. These factors might affect autonomic control through the blood–brain barrier, contributing simultaneously to both enuresis and sleep apnea.

Alternatively, renal function could link both NDDs and enuresis. Generally, SCD is known to modulate nephropathy and renal function [45]. Renal abnormalities have also been considered as an underlying cause of enuresis, through increased diuresis, free water reabsorption, and solute excretion [46]. SCD may therefore increase the risk of enuresis through renal changes. Other cohort studies of enuresis in SCD have further shown that enuresis is associated with higher lactate dehydrogenase levels [16], which in turn correlate with creatinine clearance as a potential renal insufficiency biomarker [47]. Notably, recent large-scale cohort studies also show that, among adults with NDDs and when adjusting for risk and demographic factors, there is a higher risk for chronic kidney disease than in adults without NDDs [48]. This suggests a poorly elucidated potential “kidney–brain axis” [49], which may modulate associations between NDDs and enuresis severity in individuals with SCD.

Despite the potential difference in the etiology of enuresis in SCD, treatments for enuresis may reflect guidelines for enuresis mitigation in the general population. Enuresis alarms and desmopressin are commonly prescribed in the general population [50]. In one study, intranasal desmopressin acetate for SCD patients with primary nocturnal enuresis resulted in 60 percent of patients having a complete or partial reduction in bedwetting [51]. Although this represents a small study by Figueroa et al., their results in enuretic SCD patients are comparable to past studies of the general enuretic population, which found that approximately 50 percent of patients have reduced bedwetting frequency with adherence to desmopressin [52]. Within the general population, the literature reviews have indicated that desmopressin, by itself, may be less effective than sustained long-term alarm therapy [53]. Alarm therapy, which is based primarily on principles of behavioral therapy [53], may not be affected by the different etiology of enuresis in SCD, if any. However, desmopressin, alarm therapy, and other enuresis therapies have not been comprehensively evaluated within SCD [41].

### 4.4. Pica

Our study reports lower rates of pica than previously seen in the general and SCD population studies. In particular, past studies have found that pica in SCD ranges in prevalence from 30 to 56% [22,24], which is much higher than our pica prevalence of approximately 10%. Additionally, our cohort group did not show many of the same risk factors for pica that have been found in previous studies. Past findings show pica is associated with HbSS and lower hemoglobin levels, age, and weight [22,24], none of which we saw. Other previously discussed risk factors for pica include iron deficiency and previous occurrence of stroke [23,24]. It is possible that our population was not adequately screened for pica, resulting in a small sample size that was underpowered to detect these statistical differences.

Pica is commonly reported in individuals with NDDs, such as ASD and ID [21], as well as patients with SCD [20], but few studies have looked at the confounding effect of NDDs and SCD on pica occurrence. Past studies have found that pica is generally associated with NDDs, particularly obsessive-compulsive disorders [19]. One study of a pica cohort who have both SCD and ASD found that applied behavior analysis treatment reduces pica to a near-zero rate [54].

### 4.5. Potential Etiology of Pica in SCD

The exact etiology of pica in SCD remains unclear. While iron deficiency has been proposed to cause pica [55], the iron serum levels in our sample were not significantly different. Wide-scale studies of the general population also show that pica is associated with low hemoglobin, hematocrit, and plasma zinc, although the directionality of this association was not defined [55]. Although we did not examine zinc levels, zinc deficiency is associated with pica in the general population [55]. Zinc deficiency is also common in SCD [56]. Notably, zinc deficiency in SCD has partially been attributed to reduced reabsorption of zinc in the renal tubules [56]. This suggests that widespread zinc deficiency in SCD patients predisposes them to pica [22], offering an avenue for greater research exploring the interplay between NDDs, altered kidney-mediated zinc absorption, and pica prevalence in SCD.

While there were limited significant differences between the cohorts with and without pica, we noted differences from the general population characteristics of pica. Past literature on the non-SCD population has found “ice” pica is the most common, with studies reporting 34.5% frequency, while other common items include chalk, soap, and clay [57]. In SCD, past studies have also found that dysfunctional eating patterns, while potentially being a sign of pica, can occur independently of pica [58]. While some case reports have helped to define the specifics of pica in patients with SCD [27], larger cohort studies are necessary to clarify what constitutes severe pica and the risk factors specific to SCD.

### 4.6. Limitations

Limitations include a small sample size, especially for pica, limiting the degrees of freedom, so some analyses may be underpowered to detect significant differences. Particularly, although we showed some differences in pica and enuresis occurrence on the basis of SCD type, our sample was too small for subgroup analyses. Furthermore, specific DNA mutations in patients were not available. Larger, multi-center studies are needed to confirm these associations and better understand the prevalence and risk factors across diverse populations, particularly as they pertain to specific learning difficulties and SCD-type. Some patients may have received primary or specialty care for these conditions elsewhere, and those records were not linked to the reviewed charts. Similarly, there is a potential for selection bias, as individuals with more severe symptoms or complications may be more likely to be referred, thus overreporting the prevalence of pica and enuresis. Inversely, however, given the nature of a retrospective chart review, based on diagnosed conditions, and since many of the conditions, enuresis and pica, are underrecognized, our prevalence rates may be an underestimation. Still, given that this was performed in an SCD clinic, these issues are ideally minimized and do not affect the core conclusions of our study. While our study did not investigate the role of social determinants of health in NDD, pica, and enuresis prevalence, this is an important avenue for future study. Stratifications in genotypes may aid in explaining ethnicity-dependent outcomes. Finally, the absence of a control group makes prevalence comparisons difficult for the general population.

## 5. Implications and Future Directions

Pica and enuresis commonly occur alongside one another and NDDs, demonstrating the need for increased standardized screening. Of note, the actual rate of NDDs, pica, and nocturnal enuresis may be far higher due to relatively low rates of proper developmental screening (i.e., performing full screenings for NDDs, pica, and enuresis). While the American Society of Hematology and American Academy of Pediatrics guidelines suggest surveillance and screening for neurocognitive conditions in patients with SCD, given their high-risk status, these screenings are not consistently occurring [30,59]. Given the relatively high presence of nocturnal enuresis and pica, these are likely to be readily observed by caregivers. Thus, pica or enuresis occurrence may serve as indicators for previously unknown NDD risk or elevated SCD severity. However, our chart review shows that providers offering screenings remain low; among the few charts that mention pica or enuresis in any capacity, the vast majority do not catalog specific details, including frequency, temporality, duration, and foods eaten. One potential alternative would be to increase training and knowledge for care providers to provide screenings, which may highlight previously under-reported symptoms. For pica, past studies have shown that quality improvement initiatives in a hospital system increased screening rates for pica behaviors from a sub-40% rate to a near-consistent 100% rate, suggesting the feasibility of implementing screening programs for these conditions [25]. Once screened more rigorously, providers may apply effective techniques for reducing these conditions in the general population, such as behavioral therapy for pica [54] and desmopressin or other medications for nocturnal enuresis. Furthermore, the distinct pathogenesis for these conditions in SCD, as opposed to the general population, warrants further investigation to understand and address these overlapping challenges. Given the influence of age in both pica and enuresis prevalence, a longitudinal follow-up is needed to better understand how pica, enuresis, and NDD evolve in children with SCD. Finally, future studies should also consider differences in pica and enuresis across socioeconomic and psychosocial determinants of health.

## Figures and Tables

**Table 1:** Stratifications on the basis of enuresis status in demographics, sickle cell disease severity, and medical history across sickle cell disease patients. Note: Significant results are bolded. Chi-squared test and Wilcoxon rank-sum test were used unless otherwise indicated. For nominal categories, additional post hoc tests were performed to calculate residuals, which are stated alongside the *p*-value. Column percentages are presented in the Table. Categories are not exclusive for all variables. Abbreviations: *n*, no. of study participants; SD, standard deviation; and NDD, neurodevelopmental disorder.

Factor	Group with Enuresis (*n* = 73)	Group Without Enuresis (*n* = 202)	*p*-Value
**Mean age in years when chart reviewed (SD)**	9.12 (0.57)	8.57 (0.31)	0.3654
**Sex**			0.1430
Male	42 (57.5)	96 (47.5)	
Female	31 (42.5)	106 (52.5)	
**SCD Type (%)**			**0.0030**
SS	56 (76.7)	115 (56.9)	**0.0028**
SC	7 (9.6)	64 (31.7)	**0.0002**
S Beta Thalassemia0	2 (2.7)	4 (2.0)	0.7032
S Beta Thalassemia+	8 (11.0)	19 (9.4)	0.7025
**SCD Complications (%)**			
Stroke	13 (17.8)	20 (9.9)	**0.0460**
Silent Cerebral Infarction	10 (13.7)	8 (4.0)	**0.0039**
Seizure	3 (4.1)	9 (4.5)	0.9010
Headache	48 (65.8)	99 (49.0)	**0.0140**
Pain Crises	51 (69.9)	135 (66.8)	0.6350
**History of SCD Treatments (%)**			**0.0100**
None	25 (34.3)	116 (57.4)	**0.0007**
Hydroxyurea (HU)	37 (50.7)	64 (31.7)	**0.0039**
Chronic Transfusion Therapy (CTT)	5 (6.9)	9 (4.5)	0.4255
**Other Medical Diagnoses (%)**			
Constipation	25 (34.3)	65 (32.2)	0.9270
Diarrhea	1 (1.4)	4 (2.0)	0.4380
Sleeping Status			**<0.001**
None	38 (52.1)	136 (67.3)	**0.0203**
Sleep Apnea	23 (31.5)	26 (12.9)	**0.0004**
Insomnia	9 (12.3)	12 (5.9)	0.0782
Fragmented	1 (1.4)	19 (9.4)	**0.0235**
Excessive Sleeping	2 (2.7)	9 (4.5)	0.5215
**Positive NDD (%)**	26 (35.6)	39 (19.3)	**0.0050**

**Table 2:** Stratifications on the basis of pica occurrence in demographics, sickle cell disease severity, and medical history across sickle cell disease patients. Note: Significant results are bolded. Chi-squared test and Wilcoxon rank-sum test are used unless otherwise indicated. For nominal categories, additional post hoc tests were performed to calculate residuals, which are stated alongside *p*-value. Column percentages are presented in the Table. Categories are not exclusive for all variables. Abbreviations: *n*, no. of study participants; SD, standard deviation; and NDD, neurodevelopmental disorder.

Factor	Group with Pica (*n* = 26)	Group Without Pica (*n* = 249)	*p*-Value
**Mean age in years when chart reviewed (SD)**	7.54 (0.84)	8.84 (0.29)	0.1669
**Sex**			0.6660
Male	12 (46.1)	126 (50.6)	
Female	14 (53.9)	123 (49.4)	
**SCD Type (%)**			0.0510
SS	16 (61.5)	155 (62.3)	0.9434
SC	3 (11.5)	68 (27.3)	0.0805
S Beta Thalassemia0	1 (3.9)	5 (2.0)	0.5419
S Beta Thalassemia+	6 (23.1)	21 (8.4)	**0.0169**
**SCD Complications (%)**			
Stroke	2 (7.7)	31 (12.5)	0.4770
Silent Cerebral Infarction	2 (7.7)	16 (6.4)	
Pain Crises	18 (69.2)	168 (67.5)	0.8550
**History of SCD Treatments (%)**			0.4520
None	11 (42.3)	130 (52.2)	
Hydroxyurea (HU)	14 (53.9)	87 (34.9)	
Chronic Transfusion Therapy (CTT)	0 (0)	14 (5.6)	
**Vitamin or Nutrition Deficiency**			0.1260
Vitamin D	8 (30.8)	60 (24.1)	0.4530
G6PD	0 (0)	7 (2.8)	
Vitamin C	3 (11.5)	13 (5.2)	
Lactase	0 (0)	1 (0.4)	
Copper	0 (0)	1 (0.4)	
**Other Medical Diagnoses (%)**			
Constipation	9 (34.6)	81 (32.6)	0.8850
Diarrhea	1 (3.9)	4 (1.6)	0.1250
**Abnormal Eating**			**0.0320**
None	20 (76.9)	221 (88.8)	0.0812
Poor Appetite	4 (15.4)	25 (10.0)	0.3987
High Fast-Food Frequency	2 (7.7)	2 (0.8)	**0.0052**
Overeating	0 (0)	1 (0.4)	0.7459
**Positive NDD (%)**	11 (42.3)	54 (21.7)	**0.0190**

## Data Availability

The data presented in this study are available on request from the corresponding author. The data are not publicly available due to privacy restrictions.

## Glossary

504 Plan	504 rehabilitation plan
ASH	American Society of Hematology
ADHD	Attention Deficit Hyperactivity Disorder
ASD	Autism spectrum disorder
HBB	Hemoglobin beta
HbS/β0	Hemoglobin S-β-thalassemia
HbSS	Homozygous genotypes
IEP	Individualized educational plan
ID	Intellectual disability
NDDs	Neurodevelopmental disorders
SCD	Sickle cell disease
SD	Standard deviation
